# The Role of Adipokines and Bone Marrow Adipocytes in Breast Cancer Bone Metastasis

**DOI:** 10.3390/ijms21144967

**Published:** 2020-07-14

**Authors:** Eunah Shin, Ja Seung Koo

**Affiliations:** 1 Department of Pathology, Yonsei University College of Medicine, Seoul 03722, Korea; eunahshin@yuhs.ac; 2 Department of Pathology, Yonsei University College of Medicine, Severance Hospital, 50 Yonsei-ro, Seodaemun-gu, Seoul 120-752, Korea

**Keywords:** bone marrow, adipokines, adipocyte, bone metastasis, breast cancer

## Abstract

The morbidity and mortality of breast cancer is mostly due to a distant metastasis, especially to the bone. Many factors may be responsible for bone metastasis in breast cancer, but interactions between tumor cells and other surrounding types of cells, and cytokines secreted by both, are expected to play the most important role. Bone marrow adipocyte (BMA) is one of the cell types comprising the bone, and adipokine is one of the cytokines secreted by both breast cancer cells and BMAs. These BMAs and adipokines are known to be responsible for cancer progression, and this review is focused on how BMAs and adipokines work in the process of breast cancer bone metastasis. Their potential as suppressive targets for bone metastasis is also explored in this review.

## 1. Introduction

Breast cancer is the most frequently found cancer in the female population and also one of the most prevalent causes of cancer-related deaths [1]. Despite recent advances in breast cancer treatment, it still poses an important health problem for women worldwide. The morbidity and mortality of breast cancer are mostly due to a distant metastasis, which most often occurs in the lung, liver, brain and bone [2,3]. Among these frequent sites of breast cancer metastasis, bone metastasis occurs in about two thirds of advanced breast cancer patients and leaves them with an expected survival of two to three years [4,5]. The “seed and soil hypothesis” explains the mechanism by which a certain primary cancer metastasizes to a specific organ, and according to this theory, the breast cancer cell is the “seed” and the bone is the “soil” in cases of breast cancer with bone metastasis [6]. Therefore, breast cancer cells and bone are major factors involved throughout the process of bone metastasis in breast cancer, and bone marrow adipocyte (BMA) is one of the bone factors in the bone microenvironment. Both BMAs and breast cancer cells secrete adipokines, and this review is focused on how BMAs and adipokines are involved in the process of breast cancer bone metastasis and how they can work as potential suppressive targets of bone metastasis.

## 2. Basics of Adipokines

Adipokines are metabolites, lipids, and bioactive peptides secreted by adipose tissue, and there more than 600 adipokines have been discovered to date, including batokines and lipokines [7,8,9]. Adipose tissue is composed of adipocytes, pre-adipocytes, fibroblasts, macrophages, stromal cells, endothelial cells and monocytes [10]. Thus, adipokines can be secreted by these different kinds of cells comprising adipose tissue, and most adipokines are reported to be secreted by cells other than mature adipocytes [11]. Adipokines are generally involved in appetite control, fat distribution, insulin secretion, energy expenditure, inflammation and blood pressure [12]. As for the role of adipokines in cancer, cancer cells express adipokine receptors, through which adipokines play their role in cancer cells. [13]. Adipokines can be grouped into pro-inflammatory types (leptin, tumor necrosis factor-*α* (TNF*α*), interleukin-6 (IL-6), monocyte chemoattractant protein-1 (MCP-1), haptoglobin, IL-8, IL-1B, fibrinogen, resistin, apelin, chemerin, granzyme B, visfatin, lipocalin-2, WNT1-inducible-signaling pathway protein 1 (WISP1), dipeptidyl peptidase-4 (DPP4), vascular adhesion protein-1 (VAP-1) and retinol-binding protein 4 (RBP4)) and anti-inflammatory types (adiponectin, angiopoietin 2, omentin-1, vaspin, nesfatin, secreted frizzled-related protein 5 (SFRP5) and C1q/TNF-related proteins (CTRPs)) [14,15,16]. These various adipokines can have an effect on the development of breast cancer and its growth, progression and metastasis (review [17,18]), and their representative roles in breast cancer are listed in Table 1.

## 3. Origin of Adipokines in the Process of Breast Cancer Bone Metastasis

Adipokines are variably involved in the process of breast cancer development, progression and metastasis, and the cells of origin for these adipokines are breast cancer cells and adipocytes. Breast cancer cells have been reported to secrete various adipokines, such as leptin, adiponectin, ATX, COX-2/PEG2, MMP, cathepsin K, TGF- β, IGF1 and VEGF (review [17,18,100]). Adipocytes can be subclassified into central adipocytes and local adipocytes. Central or systemic adipocytes in obese patients can undergo changes and secrete adipokines that are altered both quantitatively and qualitatively [101]. Epidemiologic data show that postmenopausal women with obesity have a higher incidence of breast cancer, and obesity is related to breast cancer prognosis [102]. It has been suggested that obesity, which is due to an increased number and size of adipocytes, is associated with breast cancer development. However, this is not the simple criteria of overweight based on BMI; rather, it is the obesity type and menopause status that have a significant impact on breast cancer development. Obesity is not correlated with breast cancer development before menopause, but hormone-positive breast cancer incidence increments every 5 years after menopause [103]. Moreover, central obesity is related to breast cancer development in pre-menopausal women [104], and it is especially related to the development of triple-negative breast cancer (TNBC) [105,106,107]. The mechanisms by which central obesity plays a role in breast cancer development are as follows: firstly, the NFκB, JAK, STAT3 and AKT pathways are activated by decreased adiponectin and increased leptin and estrogen in the leptin–adiponectin–estrogen axis; secondly, central adipose tissue induces oxidative stress by increasing the secretion of pro-inflammatory adipokines such as IL-1B, IL-6, IL-8 and TNFα; lastly, central adipose tissue increases the secretion of micro RNA (miR)s that play a role in cancer development, such as miR-23, miR-155, miR-140 and miR-302f, and decreases the secretion of miR-148b, a tumor suppressor miR (review [108]). Therefore, central adipose tissue harbors chronic inflammation and high oxidative stress, a microenvironment strongly resembling that of the tumor microenvironment. Local adipocytes can be further subdivided into breast local adipocytes and BMAs. A total of 56% of breast parenchyme consists of adipose tissue in non-lactating breasts [109] and 35% in lactating breasts [110]. Therefore, adipocytes are invariably juxtaposed with cancer cells in breast cancer, and these adipocytes are called cancer-associated adipocytes (CAAs). CAAs secrete various adipokines that can affect breast cancer progression through interactions with breast cancer cells [111]. The other local adipocytes, BMAs, comprise the bone marrow in various proportions, depending on certain conditions. They generally comprise 70% of the bone marrow volume in average adults [112], and they also secrete various adipocytokines, such as leptin, adiponectin, IL-1β, IL-6, VCAM-1, TNF-α and VEGF [113].

In addition to breast cancer cells and adipocytes, other cell types that secrete adipokines in the process of bone metastasis are bone stromal cells, e.g., osteoblasts and osteoclasts, and cancer-associated fibroblasts (CAFs), which can be differentiated from quiescent fibroblasts. Osteoblasts and osteoclasts secrete TGF-β, IL-6, IL-8, MCP-1, VEGF, IL-1B, cathepsin K, macrophage inflammatory protein 2 (MIP-2) and resistin [114], and breast cancer cells increase the secretion of IL-6, MCP-1 and IL-8 from osteoblasts [115] and also increase the secretion of IL-8, IL-6, MCP-1, MIP-2 and VEGF [116]. CAFs secrete HGF, TGF-β, CXCL12, IL-6, VEGF and MMPs [117]. Among these, CXCL12/14 and IGF-1/2 are expressed at a higher rate in TNBC with bone metastasis than in TNBC without bone metastasis, suggesting CAFs as the source of these adipokines rather than tumor cells [118].

## 4. Bone Marrow Adipocytes (BMAs)

BMAs had simply been considered one of the components of bone marrow, and only recently has their importance as multifunctional cells surfaced. Various conditions alter the proportion of BMAs within the bone marrow; older age, obesity, malnutrition, drug use and radiation tend to increase the number of adipocytes within the bone marrow [119,120,121,122]. BMAs can be subdivided into two types: inducible or regulated BMAs (rBMAs) and constitutive BMAs (cBMAs) [123]. rBMAs can be found in the proximal location of a bone and constitute the red marrow, whereas cBMAs are located in the distal portion and constitute the yellow marrow. These two BMAs differ in development, lipid saturation, gene expression and vascular density [123,124]. BMAs are derived from Sca1+, CD45- and CD31- [125] or LepR+, CD45- and CD31- bone marrow mesenchymal cells [126], which harbor bi-potent progenitor stem cell characteristics and hence can differentiate into either adipocytes or osteoblasts. Some suggest that BMAs can be phenotypically classified into two different types according to their site of origin: BMAs in long bones are of a white phenotype, whereas BMAs in the vertebrae are of a brown phenotype [127]. BMAs are involved in bone remodeling, energy regulation and insulin metabolism by secreting various adipokines, such as hormones, cytokines and fatty acids [128,129]. The secretory profile of BMAs differs from that of adipocytes in other locations; the mRNA expression of leptin and adiponectin is lower in BMAs than in extramedullary adipocytes [130,131], and TNF-α and IL-6 expression is higher in BMAs than in visceral adipocytes [130]. BMAs are also reported to have a high pro-angiogenic and pro-apoptotic profile [132]. Furthermore, BMAs carry out diverse functions by releasing various mediators, and, especially in bone remodeling, they secrete leptin and adiponectin in order to promote the differentiation of multipotent stem cells (MSC) into osteoblasts and their proliferation. However, at the same time, BMAs can secrete chemerin in order to suppress osteoblastogenesis [127]. Additionally, BMAs secrete TNF-α and RANKL to promote osteoclastogenesis [127]. BMAs differ from other peripheral tissue adipocytes in location, cellular morphology, the characteristics of the lipid droplets within the cells, and cellular function [133] (Table 2).

## 5. The Role of Adipokines in Breast Cancer Bone Metastasis

Breast cancer cells undergo following five steps when they metastasize to the bone: (1) invasion and migration, (2) intravascular infiltration and transportation, (3) extravasation, (4) adherence and arrest to the bone matrix, and (5) colonization of the cancer cells and subsequent bone destruction [134]. The first, second and third steps are common in all distant metastases of cancer, but the fourth and fifth steps are bone metastasis-specific processes.

Adipokines are involved in breast cancer progression by binding to the adipokine receptors on breast cancer cells. Firstly, there is a metastasis-associated gene among the 64 genes controlled by leptin [135], which is secreted by the adipose stromal cells. The increased secretion of leptin promotes metastasis by increasing the expression of epithelial–mesenchymal transformation (EMT)- and metastasis-associated genes (SERPINE1, MMP-2, and IL-6) in breast cancer cells [136]. MIF, which is secreted by tumor-specific T-cells, is reported to be especially involved in the pre-metastatic niche during the process of bone metastasis by means of intravasation, angiogenesis and EMT in breast cancer circulating tumor cells (CTCs) [82]. ATX–LPA signaling is involved in breast cancer metastasis, but it also promotes osteolytic bone metastasis when LPA1 is overexpressed in the MAD-BO2 breast cancer cell line [37,137]. In addition, IL-11 and IL-8, which are secreted by breast cancer cells, change the osseous environment in order to facilitate osteoclast formation and bone colonization [138,139]. IL-8, which is produced mainly by macrophages, increases osteolytic bone metastasis by activating osteoclastogenesis through the CXCR1 receptor [48]. Breast cancer with bone metastasis shows significantly increased expression of IL-11 in comparison with breast cancer without bone metastasis, and IL-11 is known to be involved in bone metastasis through the gp130/STAT3 pathway [51]. IL-11, secreted from the bone-specific metastatic breast cancer cell line (BoM-1833), promotes osteolytic bone metastasis by activating RANKL-independent osteoclastogenesis through the JAK1/STAT3 pathway [52]. Insulin-like growth factor-binding protein 2 (IGFBP2), secreted by metastatic breast cancer cells, contributes to metastatic bone colonization by interacting with IGF type-1 receptor on endothelial cells through endothelial recruitment [56]. When breast cancer cells are exposed to a stimulated bone environment, IGFBPs show a significant alteration in expression. An increased expression of IGFBP-3 in the bone microenvironment has been reported to facilitate bone metastasis by increasing TGF-β-mediated cell proliferation [140]. TGF-β from the bone matrix stimulates breast cancer cells to secrete PTHrP and IL-11 and induces bone metastasis [60,61]. This PTHrP additionally induces tumor cell proliferation and tumor cell survival by increasing bone resorption with osteoclast activation through SMAD-independent and SMAD-dependent pathways [62]. IL-6, MCP-1 and VEGF, which are secreted by osteoblasts, enable cancer cells to colonize and proliferate in the bone microenvironment [116]. Oncostatin M (OSM), a member of the IL-6 family that is secreted by breast cancer cells, facilitates osteolytic bone metastasis by activating osteoclastogenesis via the AREG autocrine pathway [69]. Pentraxin 3 (PTX3) is overexpressed in the bone metastasis of breast cancer when compared with lung, liver and brain metastasis, because PTX3 induces osteolytic bone metastasis by facilitating cancer cell migration and macrophage migration to cancer cells and increasing osteoclast formation [76]. Bone metastasis tends to occur more frequently in breast cancer that has an increased expression of IL-1B when compared with breast cancer that does not (37% vs. 5%) [141]. Breast cancer cell lines that have bone metastasis (MDA-IV) also show an increased expression of IL-1B [142], which is secreted by both breast cancer cells and osteoblasts, and it has been reported that IL-1B promotes bone metastasis by awakening breast cancer cells that are dormant within the bone [78]. VEGF from the bone matrix promotes neovascularization in breast cancer bone metastasis [143], and VEGF secreted by breast cancer cells activates osteoclasts. It is notable that the number of osteoclasts is increased in breast cancer bone metastasis tissue while the expression of VEGF receptor 1 (VEGFR1) is increased in breast cancer tissue [144]. Cathepsin K plays an important role in osteoclast-mediated bone destruction, and it is increased in breast cancer with bone metastasis [85]. Cathepsin K from breast cancer cells activates osteoclasts, which promotes osteolytic bone metastasis [85,86], and cathepsin K from the bone marrow stromal cells promotes breast cancer bone metastasis by splicing and activating SPARC in breast cancer cells [87]. When angiopoietin-2 is overexpressed in ER-positive breast cancer cells that are dormant in the bone marrow (BM) niche, it destabilizes the niche endothelium through endothelial Tie2 receptor and awakens ER-positive breast cancer cells from their dormant states, thus resulting in cancer cell growth [145]. Angiopoietin-like protein 2, secreted by breast cancer cells, increases CXCR4 expression in breast cancer, which increases cross-talk between CXCR4 and CXCL12 and, as a result, recruits breast cancer cells to the bone metastatic site [146]. Basic fibroblast growth factor (FGF) secreted by TGF-β, which originates from BM myeloid cells, activates tumor cell proliferation through the MAPK–ERK pathway by binding to FGFR1 receptors, which increases metastatic bone lesions [147].

**Figure 1:**
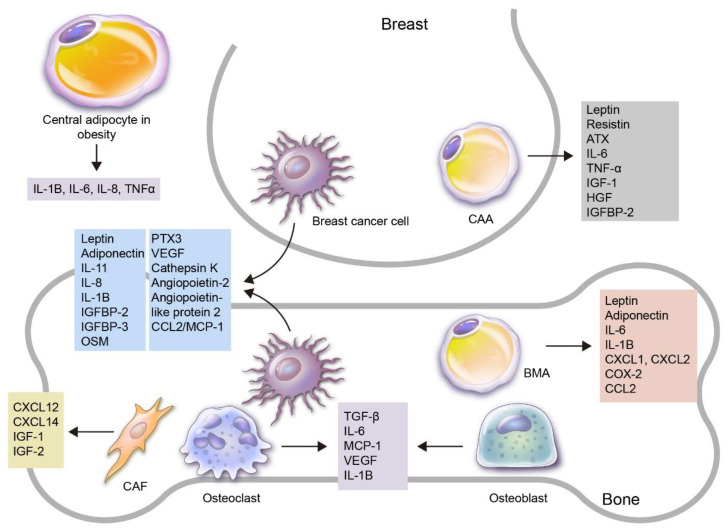
An overview of the roles of adipokines and adipocytes in the process of breast cancer bone metastasis. Various adipokines and adipocytes are involved in breast cancer development and progression to bone metastasis. Firstly, adipocytes can be subdivided into central and local adipocytes, and the former can affect breast cancer with altered adipokine secretion, especially in obese patients. Local adipocytes can be further subdivided into cancer-associated adipocytes (CAAs) in the breast and bone marrow adipocytes (BMAs) in the bone. Adipokines can be secreted by various kinds of cells: breast cancer cells secrete leptin, adiponectin, IL-1B, IL-8, IL-11, IGFBP2, IGFBP3, OSM, PTX3, VEGF, cathepsin K, angiopoietin-2, angiopoietin-like protein2 and CCL2/MCP-1, which are involved in breast cancer bone metastasis; CAAs secrete leptin, resistin, ATX, IL-6, TNF-α, IGF-1, HGF and IGFBP-2, which are involved in breast cancer progression; BMAs secrete leptin, adiponectin, IL-1B, IL-6, CXCL1, CXCL2, COX-2 and CCL2; bone stromal cells such as osteoclasts and osteoblasts secrete TGF-β, IL-6, MCP-1, VEGF, IL-1B and cathepsin K, which contribute to breast cancer bone metastasis. In addition, cancer-associated fibroblasts (CAFs) secrete CXCL12, CXCL14, IGF-1 and IGF-2.

While most adipokines promote bone metastasis (Figure 1), there are a few that suppress bone metastasis, namely CCL2/MCP-1, which inhibits bone metastasis by suppressing ICAM-1 expression and anchorage-independent tumor cell growth and cell migration [92].

## 6. Roles of BMAs in Bone Metastasis of Breast Cancer

Like other adipocytes, BMAs are also involved in breast cancer progression and metastasis, especially bone metastasis. BMAs are usually located in the endosteal surface of the diaphysis and the trabecular bone of the epiphysis and metaphysis, and bone metastasis usually occurs in the latter when active bone remodeling takes place. The bone has two physiologic niches, the endosteal niche and the vascular niche [148]. The endosteal niche is located on the surface of the trabecular and endocortical bone and is composed of osteoblasts and osteoclasts. In this endosteal niche, bone formation and bone resorption by osteoclasts occur [149]. The vascular niche is composed of endothelial cells, pericytes and smooth muscle cells, and it is involved in the recruitment of endothelial precursor cells, mesenchymal stem cells and hematopoietic stem cells [150,151]. In breast cancer patients, the physiologic niche of the bone becomes a metastatic niche through the pre-metastatic niche by means of extracellular matrix remodeling by metastatic carcinoma cells [152,153]. The metastatic niche plays a key role in determining whether the disseminated tumor cells should continue to proliferate, enter a dormant state or perish. The role of BMAs as important components of this metastatic niche has been suggested recently [79,117]. Since BMAs increase with age, their role as a metastatic niche can also increase among elderly breast cancerpatients. The following are the mechanisms by which BMAs are involved in the bone metastasis of breast cancer.

### 6.1. Adipocytokines Secreted by BMA

BMAs secrete various adipocytokines, such as leptin, adiponectin, IL-1β, IL-6, VCAM-1, TNF-α and VEGF [113]. These adipocytokines affect cancer cell biology, and, for breast cancer, IL-1β overexpression in addition to leptin is reported to induce cancer cell colonization in BM [79]. Moreover, leptin reacts with leptin receptor (LepR) on BM stem cells to promote adipogenesis by activating the Jak2/STAT3 pathway [126,154]. Additionally, leptin secreted by BMAs reacts with LepR on tumor cells and contributes to tumor progression [155,156,157]. BMAs can secrete adiponectin, which in general takes part in tumor suppression [158], and tumor patients secrete more adiponectin [121]. However, some studies report that adiponectin promotes tumor growth and migration [34,159], and these differences may result from differences in adiponectin receptor isoforms [34]. BMAs secrete a large amount of IL-6 [160], which induces EMT in cancer cells through the JAK2/STAT3 pathway [161] and increases the metastatic potential of tumor cells by promoting tumor cell survival through the PI3K/AKT pathway [162]. An interesting point is that even if tumor cells do not express IL-6 receptor (IL-6R), the soluble form of IL-6R can activate IL-6 [163]. Therefore, if BMAs secrete both IL-6 and soluble IL-6R, they can contribute to the metastatic process. IL-6 also increases the number of BMAs and forms positive feedback [164]. CXCL1 and CXCL2 from BMAs are also involved in immune modulation, working as chemoattractants for macrophages, neutrophils and CD11b+Gr1+ cells [165,166]. These immune cells express CXCL2 receptors and suppress the anti-tumor immune response [167]. In addition, the COX-2/PGE_2_ signaling axis is involved in immune modulation, resulting in chronic inflammation and immune suppression in order to facilitate tumor evasion in the immune response [168]. COX-2 and PGE_2_ overexpression is a major cause of tumor-related bone degradation in bone metastasis [169,170]. The increase in COX-2 levels induced tumor colonization and osteoclastogenesis in a breast cancer mouse model, resulting in lytic bone metastasis [171]. CCL2 from adipocytes promotes cancer progression by increasing angiogenesis [172].

### 6.2. Lipid Transfer from BMAs to Breast Cancer Cells

BMAs are a source of lipids that enable solid cancer cells to undergo cancer cell proliferation, migration and invasion [79,173]. When breast cancer cells are cultured with adipocytes in a cell line study, lipid droplets accumulate within cancer cells, and expression levels of FABP4, CD36 and perilipin 2, molecules that play roles in lipid transfer, are increased. CD36 expression is increased when breast cancer cells are cultured with BMAs [173], and exogenous lipids are transferred to breast cancer cells by CD36 to promote the growth of cancer cells [174]. In an in vivo study, the number of BMAs was increased due to increased adipogenesis in the early phase of bone metastasis; however, the number of BMAs that had an ample amount of lipid droplets decreased as the tumor progressed, supporting the lipid transfer phenomenon from BMAs to tumor cells [175]. Bone metastasis occurs more frequently in rBMA- enriched regions (proximal femur, hip and lumbar spine) rather than in cBMA-enriched regions, because rBMAs tend to transform easily depending on their microenvironment, and thus they show a flexible response to metabolic interaction with tumor cells. In support of this, the loss of BMAs can be seen in bone metastasis.

## 7. Therapeutic Targets of Adipokines and BMAs for Bone Metastasis of Breast Cancer

**Figure 2:**
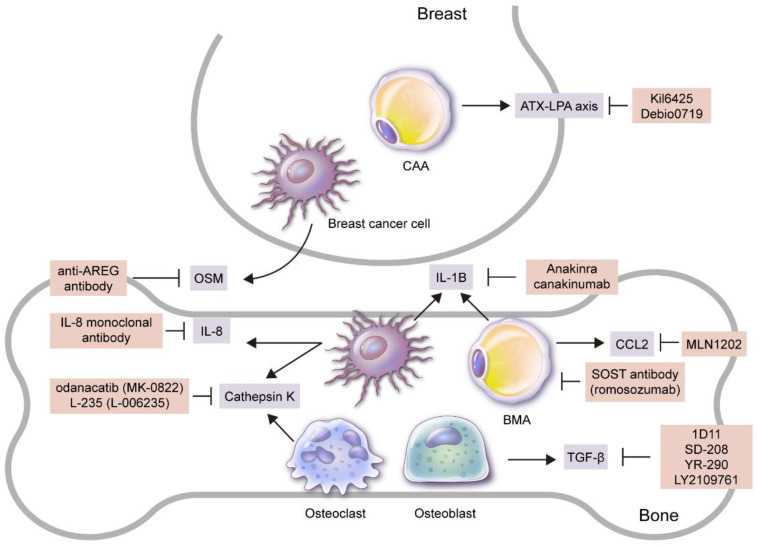
Candidates for targeted treatment of breast cancer bone metastasis through inhibition of adipokines and adipocytes. Adipokine and/or adipocyte inhibitors that have been reported to be effective in breast cancer bone metastasis treatment in preclinical and/or clinical studies are as follows: 1) SOST antibody (romosozumab) that suppresses BMA formation; 2) Ki16425 and Debio0719 secreted by CAAs that suppress ATX–LPA axis; 3) MLN1202, a monoclonal antibody to CCR2 secreted by BMAs; 4) anti-AREG antibody and IL-8 monoclonal antibody that suppress OSM secreted by breast cancer cells to inhibit osteolytic bone metastasis. Recombinant form IL-1 receptor antagonist (anakinra) that suppresses IL-1B secreted by breast cancer cells and BMAs or anti-IL-1B antibody (canakinumab) suppresses bone metastasis, as well as odanacatib (MK-0822) that suppresses cathepsin K from breast cancer cells and osteoclasts, also inhibit bone metastasis, along with L-235 (L-006235). Lastly, 1) 1D11, an anti-TGF-β antibody that represses TGF-β secreted by bone stromal cells such as osteoblasts, 2) SD-208, a small molecule TβRI-I (SD-208), 3) small molecule TGF-β inhibitor (YR-290, and 4) LY2109761 all suppress breast cancer bone metastasis.

Adipokines and BMAs play an important role in breast cancer bone metastasis through various pathways, and thus they can be effective treatment targets (Figure 2).

### 7.1. Adipokine Modulators

Adipokine modulators, namely CCL2–CCR2 axis inhibitors, can suppress cancer cells. MLN1202, a monoclonal antibody to CCR2, is currently being tested in a clinical trial for breast cancer bone metastasis [176]. Ki16425, a non-lipid competitive inhibitor of LPA1 and LAP3, decreased breast cancer bone metastasis in a mouse model [37], and Debio0719, the R-stereoisomer of Ki16425, decreased the lung and bone metastasis of breast cancer in a mouse model [177]. Cathepsin K inhibitors suppressed breast cancer-mediated osteolysis and breast cancer cell invasiveness [86], and odanacatib (MK-0822), one of the cathepsin K inhibitors, decreased bone turnover markers in a phase II clinical trial of breast cancer bone metastasis [178]. Another cathepsin K inhibitor, L-235 (L-006235), decreases breast cancer cell-induced osteolysis and tumor burden in the bone [85]. Various TGF-β inhibitors have been reported to suppress breast cancer bone metastasis. Firstly, when TGF-β type 1 receptor kinase inhibitor (TβRI-I) is transfected in the breast cancer cell line, both extensive bone metastasis and early bone metastasis are suppressed [179]. Secondly, anti-TGF-β antibody 1D11 suppresses bone loss and breast cancer bone metastasis [180], and SD-208, a small molecule TβRI-I, also suppresses breast cancer bone metastasis [181]. Other TGF-β inhibitors, YR-290 [182] and LY2109761 [183], have also been reported to suppress bone metastasis in breast cancer studies. Osteolytic bone metastasis was suppressed and survival was extended when IL-8 monoclonal antibody was injected into a mouse model using the breast cancer cell line [48]. An important factor involved in oncostatin M (OSM)-induced osteolytic bone destruction is amphiregulin (AREG), and the anti-AREG antibody is reported to suppress OSM-induced osteoclastogenesis [69]. Anakinra, a recombinant form IL-1 receptor antagonist, or canakinumab, an anti-IL-1B IgG1 antibody, decreased bone metastasis in a mouse model study of breast cancer bone metastasis [184].

### 7.2. BMA Metabolism Inhibitors

A metabolic pathway can be a treatment target because there exists a metabolic interaction between BMAs and breast cancer cells. Fatty acid released from adipocytes as a result of lipolysis is transferred to cancer cells and produces energy through mitochondrial β oxidation. This process promotes tumor progression, and, thus, the suppression of fatty acid oxidation in cancer cells can be a treatment target. Trimetazidine, a fatty acid oxidation inhibitor, has been reported to induce cancer cell apoptosis [185], and malonyl-CoA decarboxylase (MCD) inhibitor suppresses fatty acid oxidation and hence decreases human breast cancer cell proliferation by increasing malonyl-CoA level, a key enzyme that blocks fatty acids from entering mitochondria [186]. There are two ways to suppress the transfer of fatty acids from adipocytes to tumor cells: 1) an inhibitor of fatty acid-binding protein 4 (FABP 4), which is a fatty acid transporter (BMS309403), can suppress cancer cell proliferation [187], and 2) blocking a transmembrane protein CD36 that is involved in fatty acid uptake with CD36-blocking antibody can decrease breast cancer cell metastasis [188,189].

### 7.3. BMA Inhibitors

Since BMAs play an important role in breast cancer bone metastasis, the suppression of BMA formation can be an effective way to suppress bone metastasis. Sclerotin (SOST) is originally secreted by osteocytes to suppress osteoblast differentiation and hence bone formation. SOST is also involved in adipocyte generation and metabolism [190,191]; the number and size of BMAs in long bones are actually decreased when inhibited by SOST [192]. Meanwhile, SOST expression is increased in breast cancer with bone metastasis, and the SOST antibody is reported to effectively suppress bone metastasis in breast cancer [193]. A monoclonal antibody to SOST, romosozumab, is being used for osteoporosis treatment in clinical practice, and further study is needed in order to implement its use in breast cancer patients with bone metastasis [194].

## 8. Conclusions

The role of adipokines and BMAs in breast cancer bone metastasis was explored in this review. Many different kinds of adipokines are secreted by various types of cells, and those that play an important role in the process of bone metastasis are secreted by breast cancer cells, CAAs, BMAs, osteoblasts and osteoclasts. Adipokines participate in bone metastasis through angiogenesis, cancer cell migration, EMT, osteoclastogenesis, osteolysis and escape from tumor dormancy. Besides secreting adipokines, BMAs also transfer lipids to breast cancer cells as a source of energy. Since adipokines and BMAs play an important role in the process of bone metastasis, adipokine and BMA inhibitors have potential as an effective target therapy in breast cancer bone metastasis, and a few preclinical and clinical studies have implicated their therapeutic effects. However, the diversity and complexity of adipokines and BMAs may cause unexpected side effects and bring about pro-tumor effects instead of the expected anti-tumor effect, for which we need to be prepared.

## Figures and Tables

**Table 1:** Summary of the roles of adipokines in breast cancer biology.

Adipokines	Roles in Breast Cancer
Leptin	promotes breast cancer cell proliferation by JAK-STAT, ERK1/2, AKT-GSK3 and PKC-α pathways [19,20] enhances breast cancer progression through the increase of cyclin D1, and CDK2 and by the decrease of p21, p27 and p53 [20,21,22] reduces breast cancer cell apoptosis by increasing survivin and bcl-2 and decreasing caspase-9 [21,23] enhances breast cancer cell migration and invasion through ACAT2 upregulation by PI3K/AKT/SREBP2 pathway [24] elevates angiogenesis by upregulating VEGF and Notch signaling [25,26] increased leptin in adipose stromal cells leads to the expression of EMT- and metastasis-related genes [27]
Adiponectin	reduces breast cancer cell growth by inactivating p44/42 MAPK, activating AMPK pathway, and inhibiting AKT phosphorylation [28] induces breast cancer cell apoptosis through downregulating bcl-2 and upregulating p53, Bax and caspase 8 [29,30] induces autophagy related cell death by STK11/LKB1-AMPK-ULK1 pathway [31] Globular adiponectin promotes breast cancer cell invasion by autophagy [32] inhibits metastatic process by upregulation of LKB1 through AMPK-S6K axis activation [33] Globular adiponectin supports initial metastatic progression through autophagy activation [34]
Autotaxin (ATX)	induces invasion and motility of breast cancer cells by gp130/JAK/STAT3 pathway [35] LPA, an ATX receptor, stimulates breast cancer cell migration by PI3K, PAK1 and MAPK pathways [36] Overexpression of ATX–LPA signaling promotes osteolytic bone metastasis of breast cancer cells [37]
IL-6	induces EMT of breast cancer cells by STAT3 pathway [38] associated with breast cancer stem cell self-renewal through the upregulation of Sox, c-Myc and Nanog [39] increases aromatase expression and stimulates estrogen synthesis, resulting in breast cancer progression [40]
Resistin	enhances the metastatic potential of breast cancer by EMT and stemness [41] promotes breast cancer metastasis by phosphorylation of ezrin, radixin and moesin complex [42] High expression of resistin in breast cancer is associated with poor prognosis [43,44]
IL-8	IL-8 overexpression promotes cell migration via PI3K-AKT signaling pathway and EMT in triple-negative breast cancer [45] activates breast cancer-associated adipocytes (CAAs) and promotes tumorigenic effects of CAA [46] associated with early steps of breast cancer cell dissemination by adipocytes [47] activates osteoclastogenesis through CXCR1 receptor and promotes osteolytic bone metastasis in breast cancer [48]
IL-11	associated with stemness and metastasis in breast cancer [49] promotes osteoclastogenesis by sustaining the pool of osteoclast progenitor cells [50] involved in breast cancer bone metastasis through gp130/STAT3 pathway [51] activates osteoclastogenesis through JAK1/STAT3 pathway and promotes osteolytic bone metastasis [52]
IGFBP2	IGFBP2 overexpression is associated with breast cancer proliferation, invasion and migration [53] IGFBP2 overexpression is associated with breast cancer lymph node metastasis [54,55] induces metastatic bone colonization by endothelium recruitment through interaction with IGF type-I receptor [56]
TGF-β	facilitates breast cancer migration and invasion through Smad3 and ERK/Sp1 signaling pathways [57] suppresses the antitumor function of ROR1-CAR T-cells against TNBC [58] promotes breast cancer progression by TWIST expression [59] induces bone metastasis by stimulating breast cancer cells to secrete PTHrP and IL-11 [60,61] stimulates breast cancer cells to secrete PTHrP to activate osteoclasts that increase bone resorption and promote tumor cell proliferation and survival [62]
Oncostatin M (OSM)	OSM expression is correlated with breast cancer progression by JAK/STAT pathway [63] OSM expression is correlated with mesenchymal and stem cell-like differentiation in breast cancer by PI3K pathway [64] promotes metastasis in breast cancer through pre-vascular event and increased circulating tumor cells [65] High expression of OSM in breast cancer is associated with poor prognosis by estrogen receptor downregulation [66,67,68] activates osteoclastogenesis through AREG autocrine pathway to induce osteolytic bone metastasis [69]
Osteopontin (OPN)	associated with tumor cell adhesion, migration and invasion in breast cancer by binding to integrins [70,71,72] OPN overexpression is associated with lymph node metastasis and poor prognosis in breast cancer [73]
Pentraxin 3 (PTX3)	associated with stem-like features and EMT in breast cancer [74,75] increases cancer cell migration, macrophage migration to cancer cells and osteoclast formation, which result in osteolytic bone metastasis [76]
IL-1B	drives breast cancer growth and bone metastasis [77] awakens breast cancer cells which are dormant within the bones to induce bone metastasis [78] an increased expression of IL-1β induces cancer cell colonization in bone marrow [79]
MIF	promotes breast cancer cell proliferation by activation of PI3K/AKT signaling pathway [80] promotes tumor growth and metastasis by increasing recruitment of myeloid-derived suppressor cells [81] involved in pre-metastatic niche of bone metastasis process by intravasation, angiogenesis and EMT in breast cancer CTC [82]
Cathepsin K	associated with breast cancer cell proliferation and metastasis [83] expressing fibroblasts promote invasion of breast cancer cells [84] induces osteolytic bone metastasis by activating osteoclasts [85,86] involved in breast cancer bone metastasis by splicing and activating SPARC [87]
CCL2/MCP-1	CCL2/CCR2 chemokine signaling promotes breast cancer growth and invasion [88] induces tamoxifen resistance by activating PI3K/AKT/mTOR in breast cancer [89] CCL2-induced chemokine cascade promotes breast cancer metastasis by increased recruitment of metastasis-associated macrophages [90] induces the invasiveness of human breast cancer cells through upregulation of ERO1-α and MMP-9 [91] CCL2/MCP-1 suppresses the expression of ICAM-1 and anchorage-independent tumor cell growth and cell migration, hence the suppression of bone metastasis [92]
CXCL1	promotes breast cancer migration, invasion, stem cells subpopulations, EMT, or mammosphere formation [93]
VEGF	stimulates breast cancer metastasis in conjunction with cystathionine-γ-lyase [94] stimulates breast cancer cell migration by filopodia formation via NRP1/ARHGAP17/Cdc42 regulatory network [95] confers cancer stemness by Wnt/β-catenin pathway in breast cancer cells [96] promotes breast cancer progression by EMT and activation of NF-κB and β-catenin [97]
TNFα	induces trastuzumab resistance in HER2-positive breast cancer cell by MUC4 expression [98] promotes breast cancer metastasis by mesenchymal stromal cell activation via recruiting CXCR2-positive neutrophils [99]

JAK, Janus tyrosine kinase; STAT, signal transducer and activator of transcription; ERK, extracellular-signal-regulated kinase; AKT, protein kinase B; GSK, glycogen synthase kinase; PKC, protein kinase C; CDK, cyclin-dependent kinase; ACAT, Acyl-CoA: cholesterol acyltransferase; PI3K, phosphoinositide 3-kinases; SREBP2, sterol regulatory element-binding protein; VEGF, vascular endothelial growth factor; EMT, epithelial–mesenchymal transition; MAPK, mitogen activated protein kinase; AMPK, AMP-activated protein kinase; STK11, serine/threonine kinase 11; ULK1, Unc-51-like autophagy activating kinase; LKB1, liver kinase B1; S6K, S6 kinase; LPA, lysophosphatidic acid; PAK, p21-activated kinase; CXCR, C-X-C chemokine receptor; IGFBP, insulin-like growth factor-binding protein; TGF, transforming growth factor; ROR, receptor tyrosine kinase-like orphan receptor; CAR, chimeric antigen receptor; TNBC, triple-negative breast cancer; PTHrP, parathyroid hormone-related protein; AREG, amphiregulin; CTC, circulating tumor cells; MIF, macrophage migration inhibitory factor; SPARC, secreted protein acidic and rich in cysteine; CCL2/CCR2, chemokine ligand 2/chemokine receptor 2; ERO1-α, ER oxidoreductin 1-α; MMP, matrix metalloproteinase; ICAM, intercellular adhesion molecule.

**Table 2:** Difference between bone marrow adipocytes (BMAs) and peripheral tissue adipocytes.

Parameters	BMA		Peripheral Tissue Adipocyte	
	cBMA	rBMA	White	Brown
Cell shape	Spherical	Spherical	Spherical	Elliptical
Cell size	37-41μm	30-36μm	25–200 μm	15–60 μm
Lipid droplets	Unilocular	Unilocular	Unilocular and large	Multilocular and small
Location	Distal site	Proximal Central endosteal	Visceral Subcutaneous	Cervical Perirenal interscapular
Function	No response to environmental stimuli	Response to environmental stimuli	Store energy in the form of TG	Fat consumption in order to maintain body temperature

cBMA, constitutive bone marrow adipocyte; rBMA, regulated bone marrow adipocyte; UCP1, uncoupling protein 1; TG, triglyceride.

## References

[1] Ferlay J., Soerjomataram I., Dikshit R., Eser S., Mathers C., Rebelo M., Parkin D.M., Forman D., Bray F. (2015). Cancer incidence and mortality worldwide: Sources, methods and major patterns in globocan 2012. Int. J. Cancer.

[2] Weil R.J., Palmieri D.C., Bronder J.L., Stark A.M., Steeg P.S. (2005). Breast cancer metastasis to the central nervous system. Am. J. Pathol..

[3] Woodhouse E.C., Chuaqui R.F., Liotta L.A. (1997). General mechanisms of metastasis. Cancer.

[4] Fang J., Xu Q. (2015). Differences of osteoblastic bone metastases and osteolytic bone metastases in clinical features and molecular characteristics. Clin. Transl. Oncol..

[5] Kuchuk I., Hutton B., Moretto P., Ng T., Addison C.L., Clemons M. (2013). Incidence, consequences and treatment of bone metastases in breast cancer patients-experience from a single cancer centre. J. Bone Oncol..

[6] Rucci N., Teti A. (2018). Osteomimicry: How the seed grows in the soil. Calcif. Tissue Int..

[7] Lehr S., Hartwig S., Sell H. (2012). Adipokines: A treasure trove for the discovery of biomarkers for metabolic disorders. Proteomics. Clin. Appl..

[8] Rodríguez A., Becerril S., Hernández-Pardos A.W., Frühbeck G. (2020). Adipose tissue depot differences in adipokines and effects on skeletal and cardiac muscle. Curr. Opin. Pharm..

[9] Villarroya F., Cereijo R., Villarroya J., Giralt M. (2017). Brown adipose tissue as a secretory organ. Nat. Rev. Endocrinol.

[10] Geloen A., Roy P.E., Bukowiecki L.J. (1989). Regression of white adipose tissue in diabetic rats. Am. J. Physiol..

[11] Fain J.N., Madan A.K., Hiler M.L., Cheema P., Bahouth S.W. (2004). Comparison of the release of adipokines by adipose tissue, adipose tissue matrix, and adipocytes from visceral and subcutaneous abdominal adipose tissues of obese humans. Endocrinology.

[12] Bluher M., Mantzoros C.S. (2015). From leptin to other adipokines in health and disease: Facts and expectations at the beginning of the 21st century. Metab. Clin. Exp..

[13] Jarde T., Caldefie-Chezet F., Damez M., Mishellany F., Penault-Llorca F., Guillot J., Vasson M.P. (2008). Leptin and leptin receptor involvement in cancer development: A study on human primary breast carcinoma. Oncol. Rep..

[14] Barchetta I., Cimini F.A., Ciccarelli G., Baroni M.G., Cavallo M.G. (2019). Sick fat: The good and the bad of old and new circulating markers of adipose tissue inflammation. J. Endocrinol. Invest..

[15] Mancuso P., Bouchard B. (2019). The impact of aging on adipose function and adipokine synthesis. Front. Endocrinol..

[16] Spyrou N., Avgerinos K.I., Mantzoros C.S., Dalamaga M. (2018). Classic and novel adipocytokines at the intersection of obesity and cancer: Diagnostic and therapeutic strategies. Curr. Obes. Rep..

[17] Christodoulatos G.S., Spyrou N., Kadillari J., Psallida S., Dalamaga M. (2019). The role of adipokines in breast cancer: Current evidence and perspectives. Curr. Obes. Rep..

[18] Cha Y.J., Koo J.S. (2018). Adipokines as therapeutic targets in breast cancer treatment. Expert Opin. Ther. Targets.

[19] Garofalo C., Sisci D., Surmacz E. (2004). Leptin interferes with the effects of the antiestrogen ici 182,780 in mcf-7 breast cancer cells. Clin. Cancer Res. Off. J. Am. Assoc. Cancer Res..

[20] Chen C., Chang Y.C., Liu C.L., Chang K.J., Guo I.C. (2006). Leptin-induced growth of human zr-75-1 breast cancer cells is associated with up-regulation of cyclin d1 and c-myc and down-regulation of tumor suppressor p53 and p21waf1/cip1. Breast Cancer Res. Treat..

[21] Perera C.N., Spalding H.S., Mohammed S.I., Camarillo I.G. (2008). Identification of proteins secreted from leptin stimulated mcf-7 breast cancer cells: A dual proteomic approach. Exp. Biol. Med..

[22] Okumura M., Yamamoto M., Sakuma H., Kojima T., Maruyama T., Jamali M., Cooper D.R., Yasuda K. (2002). Leptin and high glucose stimulate cell proliferation in mcf-7 human breast cancer cells: Reciprocal involvement of pkc-alpha and ppar expression. Biochim. Et Biophys. Acta.

[23] Jiang H., Yu J., Guo H., Song H., Chen S. (2008). Upregulation of survivin by leptin/stat3 signaling in mcf-7 cells. Biochem. Biophys. Res. Commun..

[24] Huang Y., Jin Q., Su M., Ji F., Wang N., Zhong C., Jiang Y., Liu Y., Zhang Z., Yang J. (2017). Leptin promotes the migration and invasion of breast cancer cells by upregulating acat2. Cell Oncol (Dordr).

[25] Gonzalez-Perez R.R., Xu Y., Guo S., Watters A., Zhou W., Leibovich S.J. (2010). Leptin upregulates vegf in breast cancer via canonic and non-canonical signalling pathways and nfkappab/hif-1alpha activation. Cell. Signal..

[26] Guo S., Gonzalez-Perez R.R. (2011). Notch, il-1 and leptin crosstalk outcome (nilco) is critical for leptin-induced proliferation, migration and vegf/vegfr-2 expression in breast cancer. PLoS ONE.

[27] Bowers L.W., Rossi E.L., McDonell S.B., Doerstling S.S., Khatib S.A., Lineberger C.G., Albright J.E., Tang X., de Graffenried L.A., Hursting S.D. (2018). Leptin signaling mediates obesity-associated csc enrichment and emt in preclinical tnbc models. Mol. Cancer Res. Mcr..

[28] Dieudonne M.N., Bussiere M., Dos Santos E., Leneveu M.C., Giudicelli Y., Pecquery R. (2006). Adiponectin mediates antiproliferative and apoptotic responses in human mcf7 breast cancer cells. Biochem. Biophys. Res. Commun..

[29] Dos Santos E., Benaitreau D., Dieudonne M.N., Leneveu M.C., Serazin V., Giudicelli Y., Pecquery R. (2008). Adiponectin mediates an antiproliferative response in human mda-mb 231 breast cancer cells. Oncol. Rep..

[30] Grossmann M.E., Nkhata K.J., Mizuno N.K., Ray A., Cleary M.P. (2008). Effects of adiponectin on breast cancer cell growth and signaling. Br. J. Cancer.

[31] Chung S.J., Nagaraju G.P., Nagalingam A., Muniraj N., Kuppusamy P., Walker A., Woo J., Győrffy B., Gabrielson E., Saxena N.K. (2017). Adipoq/adiponectin induces cytotoxic autophagy in breast cancer cells through stk11/lkb1-mediated activation of the ampk-ulk1 axis. Autophagy.

[32] Falk Libby E., Liu J., Li Y.I., Lewis M.J., Demark-Wahnefried W., Hurst D.R. (2016). Globular adiponectin enhances invasion in human breast cancer cells. Oncol. Lett..

[33] Saxena N.K., Sharma D. (2010). Metastasis suppression by adiponectin: Lkb1 rises up to the challenge. Cell Adh. Migr..

[34] Libby E.F., Frost A.R., Demark-Wahnefried W., Hurst D.R. (2014). Linking adiponectin and autophagy in the regulation of breast cancer metastasis. J. Mol. Med..

[35] Azare J., Doane A., Leslie K., Chang Q., Berishaj M., Nnoli J., Mark K., Al-Ahmadie H., Gerald W., Hassimi M. (2011). Stat3 mediates expression of autotaxin in breast cancer. PLoS ONE.

[36] Du J., Sun C., Hu Z., Yang Y., Zhu Y., Zheng D., Gu L., Lu X. (2010). Lysophosphatidic acid induces mda-mb-231 breast cancer cells migration through activation of pi3k/pak1/erk signaling. PLoS ONE.

[37] Boucharaba A., Serre C.M., Grès S., Saulnier-Blache J.S., Bordet J.C., Guglielmi J., Clézardin P., Peyruchaud O. (2004). Platelet-derived lysophosphatidic acid supports the progression of osteolytic bone metastases in breast cancer. J. Clin. Investig..

[38] Gyamfi J., Lee Y.H., Eom M., Choi J. (2018). Interleukin-6/stat3 signalling regulates adipocyte induced epithelial-mesenchymal transition in breast cancer cells. Sci. Rep..

[39] Picon-Ruiz M., Pan C., Drews-Elger K., Jang K., Besser A.H., Zhao D., Morata-Tarifa C., Kim M., Ince T.A., Azzam D.J. (2016). Interactions between adipocytes and breast cancer cells stimulate cytokine production and drive src/sox2/mir-302b-mediated malignant progression. Cancer Res..

[40] Purohit A., Ghilchik M.W., Duncan L., Wang D.Y., Singh A., Walker M.M., Reed M.J. (1995). Aromatase activity and interleukin-6 production by normal and malignant breast tissues. J. Clin. Endocrinol. Metab..

[41] Avtanski D., Garcia A., Caraballo B., Thangeswaran P., Marin S., Bianco J., Lavi A., Poretsky L. (2019). Resistin induces breast cancer cells epithelial to mesenchymal transition (emt) and stemness through both adenylyl cyclase-associated protein 1 (cap1)-dependent and cap1-independent mechanisms. Cytokine.

[42] Lee J.O., Kim N., Lee H.J., Lee Y.W., Kim S.J., Park S.H., Kim H.S. (2016). Resistin, a fat-derived secretory factor, promotes metastasis of mda-mb-231 human breast cancer cells through erm activation. Sci. Rep..

[43] Dalamaga M., Sotiropoulos G., Karmaniolas K., Pelekanos N., Papadavid E., Lekka A. (2013). Serum resistin: A biomarker of breast cancer in postmenopausal women? Association with clinicopathological characteristics, tumor markers, inflammatory and metabolic parameters. Clin. Biochem..

[44] Lee Y.C., Chen Y.J., Wu C.C., Lo S., Hou M.F., Yuan S.S. (2012). Resistin expression in breast cancer tissue as a marker of prognosis and hormone therapy stratification. Gynecol. Oncol..

[45] Deng F., Weng Y., Li X., Wang T., Fan M., Shi Q. (2020). Overexpression of il-8 promotes cell migration via pi3k-akt signaling pathway and emt in triple-negative breast cancer. Pathol. Res. Pr..

[46] Al-Khalaf H.H., Al-Harbi B., Al-Sayed A., Arafah M., Tulbah A., Jarman A., Al-Mohanna F., Aboussekhra A. (2019). Interleukin-8 activates breast cancer-associated adipocytes and promotes their angiogenesis- and tumorigenesis-promoting effects. Mol. Cell. Biol..

[47] Vazquez Rodriguez G., Abrahamsson A., Jensen L.D.E., Dabrosin C. (2018). Adipocytes promote early steps of breast cancer cell dissemination via interleukin-8. Front. Immunol..

[48] Kamalakar A., Bendre M.S., Washam C.L., Fowler T.W., Carver A., Dilley J.D., Bracey J.W., Akel N.S., Margulies A.G., Skinner R.A. (2014). Circulating interleukin-8 levels explain breast cancer osteolysis in mice and humans. Bone.

[49] Samaeekia R., Adorno-Cruz V., Bockhorn J., Chang Y.F., Huang S., Prat A., Ha N., Kibria G., Huo D., Zheng H. (2017). Mir-206 inhibits stemness and metastasis of breast cancer by targeting mkl1/il11 pathway. Clin. Cancer Res. Off. J. Am. Assoc. Cancer Res..

[50] McCoy E.M., Hong H., Pruitt H.C., Feng X. (2013). Il-11 produced by breast cancer cells augments osteoclastogenesis by sustaining the pool of osteoclast progenitor cells. Bmc Cancer.

[51] Ren L., Wang X., Dong Z., Liu J., Zhang S. (2013). Bone metastasis from breast cancer involves elevated il-11 expression and the gp130/stat3 pathway. Med. Oncol..

[52] Liang M., Ma Q., Ding N., Luo F., Bai Y., Kang F., Gong X., Dong R., Dai J., Dai Q. (2019). Il-11 is essential in promoting osteolysis in breast cancer bone metastasis via rankl-independent activation of osteoclastogenesis. Cell Death Dis..

[53] Du Y., Wang P. (2019). Upregulation of miip regulates human breast cancer proliferation, invasion and migration by mediated by igfbp2. Pathol. Res. Pr..

[54] Kinsinger N.M., Dudchenko A., Wong A., Kisailus D. (2013). Synergistic effect of ph and phase in a nanocrystalline titania photocatalyst. Acs. Appl. Mater. Interfaces.

[55] Wang H., Arun B.K., Wang H., Fuller G.N., Zhang W., Middleton L.P., Sahin A.A. (2008). Igfbp2 and igfbp5 overexpression correlates with the lymph node metastasis in t1 breast carcinomas. Breast J..

[56] Png K.J., Halberg N., Yoshida M., Tavazoie S.F. (2011). A microrna regulon that mediates endothelial recruitment and metastasis by cancer cells. Nature.

[57] Zhao Y., Ma J., Fan Y., Wang Z., Tian R., Ji W., Zhang F., Niu R. (2018). Tgf-β transactivates egfr and facilitates breast cancer migration and invasion through canonical smad3 and erk/sp1 signaling pathways. Mol. Oncol..

[58] Stüber T., Monjezi R., Wallstabe L., Kühnemundt J., Nietzer S.L., Dandekar G., Wöckel A., Einsele H., Wischhusen J., Hudecek M. (2020). Inhibition of tgf-β-receptor signaling augments the antitumor function of ror1-specific car t-cells against triple-negative breast cancer. J. Immunother Cancer.

[59] Yang X., Hu J., Shi C., Dai J. (2020). Activation of tgf-β1 pathway by scube3 regulates twist1 expression and promotes breast cancer progression. Cancer Biother Radiopharm.

[60] Kang Y., Siegel P.M., Shu W., Drobnjak M., Kakonen S.M., Cordon-Cardo C., Guise T.A., Massague J. (2003). A multigenic program mediating breast cancer metastasis to bone. Cancer Cell.

[61] Yin J.J., Selander K., Chirgwin J.M., Dallas M., Grubbs B.G., Wieser R., Massague J., Mundy G.R., Guise T.A. (1999). Tgf-beta signaling blockade inhibits pthrp secretion by breast cancer cells and bone metastases development. J. Clin. Investig..

[62] Kakonen S.M., Selander K.S., Chirgwin J.M., Yin J.J., Burns S., Rankin W.A., Grubbs B.G., Dallas M., Cui Y., Guise T.A. (2002). Transforming growth factor-beta stimulates parathyroid hormone-related protein and osteolytic metastases via smad and mitogen-activated protein kinase signaling pathways. J. Biol. Chem..

[63] Lapeire L., Hendrix A., Lambein K., Van Bockstal M., Braems G., Van Den Broecke R., Limame R., Mestdagh P., Vandesompele J., Vanhove C. (2014). Cancer-associated adipose tissue promotes breast cancer progression by paracrine oncostatin m and jak/stat3 signaling. Cancer Res..

[64] West N.R., Murray J.I., Watson P.H. (2014). Oncostatin-m promotes phenotypic changes associated with mesenchymal and stem cell-like differentiation in breast cancer. Oncogene.

[65] Tawara K., Bolin C., Koncinsky J., Kadaba S., Covert H., Sutherland C., Bond L., Kronz J., Garbow J.R., Jorcyk C.L. (2018). Osm potentiates preintravasation events, increases ctc counts, and promotes breast cancer metastasis to the lung. Breast Cancer Res. Bcr.

[66] Tawara K., Scott H., Emathinger J., Wolf C., LaJoie D., Hedeen D., Bond L., Montgomery P., Jorcyk C. (2019). High expression of osm and il-6 are associated with decreased breast cancer survival: Synergistic induction of il-6 secretion by osm and il-1β. Oncotarget.

[67] West N.R., Murphy L.C., Watson P.H. (2012). Oncostatin m suppresses oestrogen receptor-α expression and is associated with poor outcome in human breast cancer. Endocr. -Relat. Cancer.

[68] Doherty M.R., Parvani J.G., Tamagno I., Junk D.J., Bryson B.L., Cheon H.J., Stark G.R., Jackson M.W. (2019). The opposing effects of interferon-beta and oncostatin-m as regulators of cancer stem cell plasticity in triple-negative breast cancer. Breast Cancer Res. Bcr.

[69] Bolin C., Tawara K., Sutherland C., Redshaw J., Aranda P., Moselhy J., Anderson R., Jorcyk C.L. (2012). Oncostatin m promotes mammary tumor metastasis to bone and osteolytic bone degradation. Genes Cancer.

[70] Zhao H., Chen Q., Alam A., Cui J., Suen K.C., Soo A.P., Eguchi S., Gu J., Ma D. (2018). The role of osteopontin in the progression of solid organ tumour. Cell Death Dis..

[71] Noti J.D. (2000). Adherence to osteopontin via alphavbeta3 suppresses phorbol ester-mediated apoptosis in mcf-7 breast cancer cells that overexpress protein kinase c-alpha. Int. J. Oncol..

[72] Tuck A.B., Elliott B.E., Hota C., Tremblay E., Chambers A.F. (2000). Osteopontin-induced, integrin-dependent migration of human mammary epithelial cells involves activation of the hepatocyte growth factor receptor (met). J. Cell. Biochem..

[73] Xu Y.Y., Zhang Y.Y., Lu W.F., Mi Y.J., Chen Y.Q. (2015). Prognostic value of osteopontin expression in breast cancer: A meta-analysis. Mol. Clin. Oncol..

[74] Kampo S., Ahmmed B., Zhou T., Owusu L., Anabah T.W., Doudou N.R., Kuugbee E.D., Cui Y., Lu Z., Yan Q. (2019). Scorpion venom analgesic peptide, bmk agap inhibits stemness, and epithelial-mesenchymal transition by down-regulating ptx3 in breast cancer. Front. Oncol..

[75] Thomas C., Henry W., Cuiffo B.G., Collmann A.Y., Marangoni E., Benhamo V., Bhasin M.K., Fan C., Fuhrmann L., Baldwin A.S. (2017). Pentraxin-3 is a pi3k signaling target that promotes stem cell-like traits in basal-like breast cancers. Sci. Signal..

[76] Choi B., Lee E.J., Song D.H., Yoon S.C., Chung Y.H., Jang Y., Kim S.M., Song Y., Kang S.W., Yoon S.Y. (2014). Elevated pentraxin 3 in bone metastatic breast cancer is correlated with osteolytic function. Oncotarget.

[77] Holen I., Lefley D.V., Francis S.E., Rennicks S., Bradbury S., Coleman R.E., Ottewell P. (2016). Il-1 drives breast cancer growth and bone metastasis in vivo. Oncotarget.

[78] Sosnoski D.M., Norgard R.J., Grove C.D., Foster S.J., Mastro A.M. (2015). Dormancy and growth of metastatic breast cancer cells in a bone-like microenvironment. Clin. Exp. Metastasis.

[79] Templeton Z.S., Lie W.R., Wang W., Rosenberg-Hasson Y., Alluri R.V., Tamaresis J.S., Bachmann M.H., Lee K., Maloney W.J., Contag C.H. (2015). Breast cancer cell colonization of the human bone marrow adipose tissue niche. Neoplasia.

[80] Lue H., Thiele M., Franz J., Dahl E., Speckgens S., Leng L., Fingerle-Rowson G., Bucala R., Lüscher B., Bernhagen J. (2007). Macrophage migration inhibitory factor (mif) promotes cell survival by activation of the akt pathway and role for csn5/jab1 in the control of autocrine mif activity. Oncogene.

[81] Simpson K.D., Templeton D.J., Cross J.V. (2012). Macrophage migration inhibitory factor promotes tumor growth and metastasis by inducing myeloid-derived suppressor cells in the tumor microenvironment. J. Immunol..

[82] Martinez L.M., Vallone V.B., Labovsky V., Choi H., Hofer E.L., Feldman L., Bordenave R.H., Batagelj E., Dimase F., Villafane A.R. (2014). Changes in the peripheral blood and bone marrow from untreated advanced breast cancer patients that are associated with the establishment of bone metastases. Clin. Exp. Metastasis.

[83] Gu X., Peng Y., Zhao Y., Liang X., Tang Y., Liu J. (2019). A novel derivative of artemisinin inhibits cell proliferation and metastasis via down-regulation of cathepsin k in breast cancer. Eur J. Pharm..

[84] Kleer C.G., Bloushtain-Qimron N., Chen Y.H., Carrasco D., Hu M., Yao J., Kraeft S.K., Collins L.C., Sabel M.S., Argani P. (2008). Epithelial and stromal cathepsin k and cxcl14 expression in breast tumor progression. Clin. Cancer Res. Off. J. Am. Assoc. Cancer Res..

[85] Duong L.T., Wesolowski G.A., Leung P., Oballa R., Pickarski M. (2014). Efficacy of a cathepsin k inhibitor in a preclinical model for prevention and treatment of breast cancer bone metastasis. Mol. Cancer.

[86] Le Gall C., Bellahcene A., Bonnelye E., Gasser J.A., Castronovo V., Green J., Zimmermann J., Clezardin P. (2007). A cathepsin k inhibitor reduces breast cancer induced osteolysis and skeletal tumor burden. Cancer Res..

[87] Podgorski I., Linebaugh B.E., Koblinski J.E., Rudy D.L., Herroon M.K., Olive M.B., Sloane B.F. (2009). Bone marrow-derived cathepsin k cleaves sparc in bone metastasis. Am. J. Pathol..

[88] Brummer G., Fang W., Smart C., Zinda B., Alissa N., Berkland C., Miller D., Cheng N. (2020). Ccr2 signaling in breast carcinoma cells promotes tumor growth and invasion by promoting ccl2 and suppressing cd154 effects on the angiogenic and immune microenvironments. Oncogene.

[89] Li D., Ji H., Niu X., Yin L., Wang Y., Gu Y., Wang J., Zhou X., Zhang H., Zhang Q. (2020). Tumor-associated macrophages secrete cc-chemokine ligand 2 and induce tamoxifen resistance by activating pi3k/akt/mtor in breast cancer. Cancer Sci..

[90] Kitamura T., Qian B.Z., Soong D., Cassetta L., Noy R., Sugano G., Kato Y., Li J., Pollard J.W. (2015). Ccl2-induced chemokine cascade promotes breast cancer metastasis by enhancing retention of metastasis-associated macrophages. J. Exp. Med..

[91] Lee S., Lee E., Ko E., Ham M., Lee H.M., Kim E.S., Koh M., Lim H.K., Jung J., Park S.Y. (2018). Tumor-associated macrophages secrete ccl2 and induce the invasive phenotype of human breast epithelial cells through upregulation of ero1-α and mmp-9. Cancer Lett..

[92] Takahashi M., Miyazaki H., Furihata M., Sakai H., Konakahara T., Watanabe M., Okada T. (2009). Chemokine ccl2/mcp-1 negatively regulates metastasis in a highly bone marrow-metastatic mouse breast cancer model. Clin. Exp. Metastasis.

[93] Wang N., Zheng Y., Gu J., Cai Y., Wang S., Zhang F., Chen J., Situ H., Lin Y., Wang Z. (2017). Network-pharmacology-based validation of tams/cxcl-1 as key mediator of xiaopi formula preventing breast cancer development and metastasis. Sci. Rep..

[94] Wang L., Shi H., Liu Y., Zhang W., Duan X., Li M., Shi X., Wang T. (2019). Cystathionine-γ-lyase promotes the metastasis of breast cancer via the vegf signaling pathway. Int. J. Oncol..

[95] Kiso M., Tanaka S., Saji S., Toi M., Sato F. (2018). Long isoform of vegf stimulates cell migration of breast cancer by filopodia formation via nrp1/arhgap17/cdc42 regulatory network. Int. J. Cancer.

[96] Zhang L., Wang H., Li C., Zhao Y., Wu L., Du X., Han Z. (2017). Vegf-a/neuropilin 1 pathway confers cancer stemness via activating wnt/β-catenin axis in breast cancer cells. Cell Physiol. Biochem..

[97] Luo M., Hou L., Li J., Shao S., Huang S., Meng D., Liu L., Feng L., Xia P., Qin T. (2016). Vegf/nrp-1axis promotes progression of breast cancer via enhancement of epithelial-mesenchymal transition and activation of nf-κb and β-catenin. Cancer Lett..

[98] Mercogliano M.F., De Martino M., Venturutti L., Rivas M.A., Proietti C.J., Inurrigarro G., Frahm I., Allemand D.H., Deza E.G., Ares S. (2017). Tnfα-induced mucin 4 expression elicits trastuzumab resistance in her2-positive breast cancer. Clin. Cancer Res. Off. J. Am. Assoc. Cancer Res..

[99] Yu P.F., Huang Y., Han Y.Y., Lin L.Y., Sun W.H., Rabson A.B., Wang Y., Shi Y.F. (2017). Tnfα-activated mesenchymal stromal cells promote breast cancer metastasis by recruiting cxcr2(+) neutrophils. Oncogene.

[100] Brook N., Brook E., Dharmarajan A., Dass C.R., Chan A. (2018). Breast cancer bone metastases: Pathogenesis and therapeutic targets. Int. J. Biochem. Cell Biol..

[101] Khan S., Shukla S., Sinha S., Meeran S.M. (2013). Role of adipokines and cytokines in obesity-associated breast cancer: Therapeutic targets. Cytokine Growth Factor Rev..

[102] Chan D.S., Vieira A.R., Aune D., Bandera E.V., Greenwood D.C., McTiernan A., Navarro Rosenblatt D., Thune I., Vieira R., Norat T. (2014). Body mass index and survival in women with breast cancer-systematic literature review and meta-analysis of 82 follow-up studies. Ann. Oncol. Off. J. Eur. Soc. Med. Oncol..

[103] Chen M.J., Wu W.Y., Yen A.M., Fann J.C., Chen S.L., Chiu S.Y., Chen H.H., Chiou S.T. (2016). Body mass index and breast cancer: Analysis of a nation-wide population-based prospective cohort study on 1 393 985 taiwanese women. Int. J. Obes..

[104] Harvie M., Hooper L., Howell A.H. (2003). Central obesity and breast cancer risk: A systematic review. Obes. Rev. Off. J. Int. Assoc. Study Obes..

[105] Pierobon M., Frankenfeld C.L. (2013). Obesity as a risk factor for triple-negative breast cancers: A systematic review and meta-analysis. Breast Cancer Res. Treat..

[106] Godinho-Mota J.C.M., Martins K.A., Vaz-Gonçalves L., Mota J.F., Soares L.R., Freitas-Junior R. (2018). Visceral adiposity increases the risk of breast cancer: A case-control study. Nutr. Hosp..

[107] Wang F., Liu L., Cui S., Tian F., Fan Z., Geng C., Cao X., Yang Z., Wang X., Liang H. (2017). Distinct effects of body mass index and waist/hip ratio on risk of breast cancer by joint estrogen and progestogen receptor status: Results from a case-control study in northern and eastern china and implications for chemoprevention. Oncologist.

[108] Zimta A.A., Tigu A.B., Muntean M., Cenariu D., Slaby O., Berindan-Neagoe I. (2019). Molecular links between central obesity and breast cancer. Int. J. Mol. Sci..

[109] Vandeweyer E., Hertens D. (2002). Quantification of glands and fat in breast tissue: An experimental determination. Ann. Anat. Anat. Anz. Off. Organ. Anat. Ges..

[110] Ramsay D.T., Kent J.C., Hartmann R.A., Hartmann P.E. (2005). Anatomy of the lactating human breast redefined with ultrasound imaging. J. Anat..

[111] Bochet L., Lehuede C., Dauvillier S., Wang Y.Y., Dirat B., Laurent V., Dray C., Guiet R., Maridonneau-Parini I., Le Gonidec S. (2013). Adipocyte-derived fibroblasts promote tumor progression and contribute to the desmoplastic reaction in breast cancer. Cancer Res..

[112] Fazeli P.K., Horowitz M.C., MacDougald O.A., Scheller E.L., Rodeheffer M.S., Rosen C.J., Klibanski A. (2013). Marrow fat and bone--new perspectives. J. Clin. Endocrinol. Metab..

[113] Caers J., Deleu S., Belaid Z., De Raeve H., Van Valckenborgh E., De Bruyne E., Defresne M.P., Van Riet I., Van Camp B., Vanderkerken K. (2007). Neighboring adipocytes participate in the bone marrow microenvironment of multiple myeloma cells. Leukemia.

[114] Xie C., Chen Q. (2019). Adipokines: New therapeutic target for osteoarthritis?. Curr. Rheumatol. Rep..

[115] Kinder M., Chislock E., Bussard K.M., Shuman L., Mastro A.M. (2008). Metastatic breast cancer induces an osteoblast inflammatory response. Exp. Cell Res..

[116] Bussard K.M., Venzon D.J., Mastro A.M. (2010). Osteoblasts are a major source of inflammatory cytokines in the tumor microenvironment of bone metastatic breast cancer. J. Cell. Biochem..

[117] Haider M.T., Smit D.J., Taipaleenmäki H. (2020). The endosteal niche in breast cancer bone metastasis. Front. Oncol..

[118] Zhang X.H., Jin X., Malladi S., Zou Y., Wen Y.H., Brogi E., Smid M., Foekens J.A., Massagué J. (2013). Selection of bone metastasis seeds by mesenchymal signals in the primary tumor stroma. Cell.

[119] Berry R., Rodeheffer M.S., Rosen C.J., Horowitz M.C. (2015). Adipose tissue residing progenitors (adipocyte lineage progenitors and adipose derived stem cells (adsc). Curr. Mol. Biol. Rep..

[120] Georgiou K.R., Hui S.K., Xian C.J. (2012). Regulatory pathways associated with bone loss and bone marrow adiposity caused by aging, chemotherapy, glucocorticoid therapy and radiotherapy. Am. J. Stem Cells.

[121] Cawthorn W.P., Scheller E.L., Learman B.S., Parlee S.D., Simon B.R., Mori H., Ning X., Bree A.J., Schell B., Broome D.T. (2014). Bone marrow adipose tissue is an endocrine organ that contributes to increased circulating adiponectin during caloric restriction. Cell Metab..

[122] Berendsen A.D., Olsen B.R. (2014). Osteoblast-adipocyte lineage plasticity in tissue development, maintenance and pathology. Cell. Mol. Life Sci. Cmls.

[123] Scheller E.L., Doucette C.R., Learman B.S., Cawthorn W.P., Khandaker S., Schell B., Wu B., Ding S.Y., Bredella M.A., Fazeli P.K. (2015). Region-specific variation in the properties of skeletal adipocytes reveals regulated and constitutive marrow adipose tissues. Nat. Commun..

[124] Roche B., David V., Vanden-Bossche A., Peyrin F., Malaval L., Vico L., Lafage-Proust M.H. (2012). Structure and quantification of microvascularisation within mouse long bones: What and how should we measure?. Bone.

[125] Ambrosi T.H., Scialdone A., Graja A., Gohlke S., Jank A.M., Bocian C., Woelk L., Fan H., Logan D.W., Schurmann A. (2017). Adipocyte accumulation in the bone marrow during obesity and aging impairs stem cell-based hematopoietic and bone regeneration. Cell Stem Cell.

[126] Yue R., Zhou B.O., Shimada I.S., Zhao Z., Morrison S.J. (2016). Leptin receptor promotes adipogenesis and reduces osteogenesis by regulating mesenchymal stromal cells in adult bone marrow. Cell Stem Cell.

[127] Hardouin P., Rharass T., Lucas S. (2016). Bone marrow adipose tissue: To be or not to be a typical adipose tissue?. Front. Endocrinol..

[128] Roodman G.D. (2012). Genes associate with abnormal bone cell activity in bone metastasis. Cancer Metastasis Rev..

[129] Paula F.J., Rosen C.J. (2010). Obesity, diabetes mellitus and last but not least, osteoporosis. Arq. Bras. De Endocrinol. E Metabol..

[130] Liu L.F., Shen W.J., Ueno M., Patel S., Kraemer F.B. (2011). Characterization of age-related gene expression profiling in bone marrow and epididymal adipocytes. Bmc Genom..

[131] Poloni A., Maurizi G., Serrani F., Mancini S., Zingaretti M.C., Frontini A., Cinti S., Olivieri A., Leoni P. (2013). Molecular and functional characterization of human bone marrow adipocytes. Exp. Hematol..

[132] Gasparrini M., Rivas D., Elbaz A., Duque G. (2009). Differential expression of cytokines in subcutaneous and marrow fat of aging c57bl/6j mice. Exp. Gerontol..

[133] Boroumand P., Klip A. (2020). Bone marrow adipose cells - cellular interactions and changes with obesity. J. Cell Sci..

[134] Fidler I.J. (2003). The pathogenesis of cancer metastasis: The ‘seed and soil’ hypothesis revisited. Nat. Rev. Cancer.

[135] Perera C.N., Chin H.G., Duru N., Camarillo I.G. (2008). Leptin-regulated gene expression in mcf-7 breast cancer cells: Mechanistic insights into leptin-regulated mammary tumor growth and progression. J. Endocrinol..

[136] Strong A.L., Ohlstein J.F., Biagas B.A., Rhodes L.V., Pei D.T., Tucker H.A., Llamas C., Bowles A.C., Dutreil M.F., Zhang S. (2015). Leptin produced by obese adipose stromal/stem cells enhances proliferation and metastasis of estrogen receptor positive breast cancers. Breast Cancer Res. Bcr.

[137] Boucharaba A., Serre C.M., Guglielmi J., Bordet J.C., Clezardin P., Peyruchaud O. (2006). The type 1 lysophosphatidic acid receptor is a target for therapy in bone metastases. Proc. Natl. Acad. Sci. United States Am..

[138] Bendre M.S., Gaddy-Kurten D., Mon-Foote T., Akel N.S., Skinner R.A., Nicholas R.W., Suva L.J. (2002). Expression of interleukin 8 and not parathyroid hormone-related protein by human breast cancer cells correlates with bone metastasis in vivo. Cancer Res..

[139] Kang Y., He W., Tulley S., Gupta G.P., Serganova I., Chen C.R., Manova-Todorova K., Blasberg R., Gerald W.L., Massague J. (2005). Breast cancer bone metastasis mediated by the smad tumor suppressor pathway. Proc. Natl. Acad. Sci. United States Am..

[140] Giles E.D., Singh G. (2003). Role of insulin-like growth factor binding proteins (igfbps) in breast cancer proliferation and metastasis. Clin. Exp. Metastasis.

[141] Coleman R.E., Marshall H., Cameron D., Dodwell D., Burkinshaw R., Keane M., Gil M., Houston S.J., Grieve R.J., Barrett-Lee P.J. (2011). Breast-cancer adjuvant therapy with zoledronic acid. New Engl. J. Med..

[142] Nutter F., Holen I., Brown H.K., Cross S.S., Evans C.A., Walker M., Coleman R.E., Westbrook J.A., Selby P.J., Brown J.E. (2014). Different molecular profiles are associated with breast cancer cell homing compared with colonisation of bone: Evidence using a novel bone-seeking cell line. Endocr. -Relat. Cancer.

[143] Lee S., Jilani S.M., Nikolova G.V., Carpizo D., Iruela-Arispe M.L. (2005). Processing of vegf-a by matrix metalloproteinases regulates bioavailability and vascular patterning in tumors. J. Cell Biol..

[144] Aldridge S.E., Lennard T.W., Williams J.R., Birch M.A. (2005). Vascular endothelial growth factor acts as an osteolytic factor in breast cancer metastases to bone. Br. J. Cancer.

[145] Han H.H., Kim B.G., Lee J.H., Kang S., Kim J.E., Cho N.H. (2016). Angiopoietin-2 promotes er+ breast cancer cell survival in bone marrow niche. Endocr. -Relat. Cancer.

[146] Masuda T., Endo M., Yamamoto Y., Odagiri H., Kadomatsu T., Nakamura T., Tanoue H., Ito H., Yugami M., Miyata K. (2015). Angptl2 increases bone metastasis of breast cancer cells through enhancing cxcr4 signaling. Sci. Rep..

[147] Meng X., Vander Ark A., Lee P., Hostetter G., Bhowmick N.A., Matrisian L.M., Williams B.O., Miranti C.K., Li X. (2016). Myeloid-specific tgf-beta signaling in bone promotes basic-fgf and breast cancer bone metastasis. Oncogene.

[148] Méndez-Ferrer S., Michurina T.V., Ferraro F., Mazloom A.R., Macarthur B.D., Lira S.A., Scadden D.T., Ma’ayan A., Enikolopov G.N., Frenette P.S. (2010). Mesenchymal and haematopoietic stem cells form a unique bone marrow niche. Nature.

[149] Sims N.A., Martin T.J. (2015). Coupling signals between the osteoclast and osteoblast: How are messages transmitted between these temporary visitors to the bone surface?. Front. Endocrinol..

[150] Butler J.M., Kobayashi H., Rafii S. (2010). Instructive role of the vascular niche in promoting tumour growth and tissue repair by angiocrine factors. Nat. Rev. Cancer.

[151] Park D., Sykes D.B., Scadden D.T. (2012). The hematopoietic stem cell niche. Front. Biosci..

[152] Kaplan R.N., Riba R.D., Zacharoulis S., Bramley A.H., Vincent L., Costa C., MacDonald D.D., Jin D.K., Shido K., Kerns S.A. (2005). Vegfr1-positive haematopoietic bone marrow progenitors initiate the pre-metastatic niche. Nature.

[153] Malanchi I., Santamaria-Martínez A., Susanto E., Peng H., Lehr H.A., Delaloye J.F., Huelsken J. (2011). Interactions between cancer stem cells and their niche govern metastatic colonization. Nature.

[154] Zhou B.O., Yu H., Yue R., Zhao Z., Rios J.J., Naveiras O., Morrison S.J. (2017). Bone marrow adipocytes promote the regeneration of stem cells and haematopoiesis by secreting scf. Nat. Cell Biol..

[155] Snoussi K., Strosberg A.D., Bouaouina N., Ben Ahmed S., Helal A.N., Chouchane L. (2006). Leptin and leptin receptor polymorphisms are associated with increased risk and poor prognosis of breast carcinoma. Bmc Cancer.

[156] Jarde T., Perrier S., Vasson M.P., Caldefie-Chezet F. (2011). Molecular mechanisms of leptin and adiponectin in breast cancer. Eur. J. Cancer.

[157] Kumar J., Fang H., McCulloch D.R., Crowley T., Ward A.C. (2017). Leptin receptor signaling via janus kinase 2/signal transducer and activator of transcription 3 impacts on ovarian cancer cell phenotypes. Oncotarget.

[158] Dalamaga M., Diakopoulos K.N., Mantzoros C.S. (2012). The role of adiponectin in cancer: A review of current evidence. Endocr. Rev..

[159] Jia Z., Liu Y., Cui S. (2014). Adiponectin induces breast cancer cell migration and growth factor expression. Cell Biochem. Biophys..

[160] Laharrague P., Fontanilles A.M., Tkaczuk J., Corberand J.X., Penicaud L., Casteilla L. (2000). Inflammatory/haematopoietic cytokine production by human bone marrow adipocytes. Eur. Cytokine Netw..

[161] Wang L., Cao L., Wang H., Liu B., Zhang Q., Meng Z., Wu X., Zhou Q., Xu K. (2017). Cancer-associated fibroblasts enhance metastatic potential of lung cancer cells through il-6/stat3 signaling pathway. Oncotarget.

[162] Tu Y., Gardner A., Lichtenstein A. (2000). The phosphatidylinositol 3-kinase/akt kinase pathway in multiple myeloma plasma cells: Roles in cytokine-dependent survival and proliferative responses. Cancer Res..

[163] Knupfer H., Preiss R. (2008). Sil-6r: More than an agonist?. Immunol. Cell Biol..

[164] Fan Y., Hanai J.I., Le P.T., Bi R., Maridas D., DeMambro V., Figueroa C.A., Kir S., Zhou X., Mannstadt M. (2017). Parathyroid hormone directs bone marrow mesenchymal cell fate. Cell Metab..

[165] De Filippo K., Dudeck A., Hasenberg M., Nye E., van Rooijen N., Hartmann K., Gunzer M., Roers A., Hogg N. (2013). Mast cell and macrophage chemokines cxcl1/cxcl2 control the early stage of neutrophil recruitment during tissue inflammation. Blood.

[166] Acharyya S., Oskarsson T., Vanharanta S., Malladi S., Kim J., Morris P.G., Manova-Todorova K., Leversha M., Hogg N., Seshan V.E. (2012). A cxcl1 paracrine network links cancer chemoresistance and metastasis. Cell.

[167] Gabrilovich D.I., Nagaraj S. (2009). Myeloid-derived suppressor cells as regulators of the immune system. Nat. Reviews. Immunol..

[168] Kalinski P. (2012). Regulation of immune responses by prostaglandin e2. J. Immunol..

[169] Safina A., Sotomayor P., Limoge M., Morrison C., Bakin A.V. (2011). Tak1-tab2 signaling contributes to bone destruction by breast carcinoma cells. Mol. Cancer Res. Mcr.

[170] Singh B., Berry J.A., Shoher A., Ayers G.D., Wei C., Lucci A. (2007). Cox-2 involvement in breast cancer metastasis to bone. Oncogene.

[171] Li Z., Schem C., Shi Y.H., Medina D., Zhang M. (2008). Increased cox2 expression enhances tumor-induced osteoclastic lesions in breast cancer bone metastasis. Clin. Exp. Metastasis.

[172] Arendt L.M., McCready J., Keller P.J., Baker D.D., Naber S.P., Seewaldt V., Kuperwasser C. (2013). Obesity promotes breast cancer by ccl2-mediated macrophage recruitment and angiogenesis. Cancer Res..

[173] Herroon M.K., Rajagurubandara E., Hardaway A.L., Powell K., Turchick A., Feldmann D., Podgorski I. (2013). Bone marrow adipocytes promote tumor growth in bone via fabp4-dependent mechanisms. Oncotarget.

[174] Zhao J., Zhi Z., Wang C., Xing H., Song G., Yu X., Zhu Y., Wang X., Zhang X., Di Y. (2017). Exogenous lipids promote the growth of breast cancer cells via cd36. Oncol. Rep..

[175] Wang J., Chen G.L., Cao S., Zhao M.C., Liu Y.Q., Chen X.X., Qian C. (2017). Adipogenic niches for melanoma cell colonization and growth in bone marrow. Lab. Investig. A J. Tech. Methods Pathol..

[176] S0916, mln1202 in Treating Patients with Bone Metastases. Nct01015560. www.Clinicaltrials.gov.

[177] David M., Ribeiro J., Descotes F., Serre C.M., Barbier M., Murone M., Clezardin P., Peyruchaud O. (2012). Targeting lysophosphatidic acid receptor type 1 with debio 0719 inhibits spontaneous metastasis dissemination of breast cancer cells independently of cell proliferation and angiogenesis. Int. J. Oncol..

[178] Jensen A.B., Wynne C., Ramirez G., He W., Song Y., Berd Y., Wang H., Mehta A., Lombardi A. (2010). The cathepsin k inhibitor odanacatib suppresses bone resorption in women with breast cancer and established bone metastases: Results of a 4-week, double-blind, randomized, controlled trial. Clin. Breast Cancer.

[179] Bandyopadhyay A., Agyin J.K., Wang L., Tang Y., Lei X., Story B.M., Cornell J.E., Pollock B.H., Mundy G.R., Sun L.Z. (2006). Inhibition of pulmonary and skeletal metastasis by a transforming growth factor-beta type i receptor kinase inhibitor. Cancer Res..

[180] Biswas S., Nyman J.S., Alvarez J., Chakrabarti A., Ayres A., Sterling J., Edwards J., Rana T., Johnson R., Perrien D.S. (2011). Anti-transforming growth factor ss antibody treatment rescues bone loss and prevents breast cancer metastasis to bone. PLoS ONE.

[181] Dunn L.K., Mohammad K.S., Fournier P.G., McKenna C.R., Davis H.W., Niewolna M., Peng X.H., Chirgwin J.M., Guise T.A. (2009). Hypoxia and tgf-beta drive breast cancer bone metastases through parallel signaling pathways in tumor cells and the bone microenvironment. PLoS ONE.

[182] Fang Y., Chen Y., Yu L., Zheng C., Qi Y., Li Z., Yang Z., Zhang Y., Shi T., Luo J. (2013). Inhibition of breast cancer metastases by a novel inhibitor of tgfbeta receptor 1. J. Natl. Cancer Inst..

[183] Korpal M., Yan J., Lu X., Xu S., Lerit D.A., Kang Y. (2009). Imaging transforming growth factor-beta signaling dynamics and therapeutic response in breast cancer bone metastasis. Nat. Med..

[184] Tulotta C., Lefley D.V., Freeman K., Gregory W.M., Hanby A.M., Heath P.R., Nutter F., Wilkinson J.M., Spicer-Hadlington A.R., Liu X. (2019). Endogenous production of il1b by breast cancer cells drives metastasis and colonization of the bone microenvironment. Clin. Cancer Res. Off. J. Am. Assoc. Cancer Res..

[185] Andela V.B., Altuwaijri S., Wood J., Rosier R.N. (2005). Inhibition of beta-oxidative respiration is a therapeutic window associated with the cancer chemo-preventive activity of ppargamma agonists. Febs Lett..

[186] Zhou W., Tu Y., Simpson P.J., Kuhajda F.P. (2009). Malonyl-coa decarboxylase inhibition is selectively cytotoxic to human breast cancer cells. Oncogene.

[187] Nieman K.M., Kenny H.A., Penicka C.V., Ladanyi A., Buell-Gutbrod R., Zillhardt M.R., Romero I.L., Carey M.S., Mills G.B., Hotamisligil G.S. (2011). Adipocytes promote ovarian cancer metastasis and provide energy for rapid tumor growth. Nat. Med..

[188] Pascual G., Avgustinova A., Mejetta S., Martin M., Castellanos A., Attolini C.S., Berenguer A., Prats N., Toll A., Hueto J.A. (2017). Targeting metastasis-initiating cells through the fatty acid receptor cd36. Nature.

[189] Ladanyi A., Mukherjee A., Kenny H.A., Johnson A., Mitra A.K., Sundaresan S., Nieman K.M., Pascual G., Benitah S.A., Montag A. (2018). Adipocyte-induced cd36 expression drives ovarian cancer progression and metastasis. Oncogene.

[190] Ukita M., Yamaguchi T., Ohata N., Tamura M. (2016). Sclerostin enhances adipocyte differentiation in 3t3-l1 cells. J. Cell. Biochem..

[191] Kim S.P., Frey J.L., Li Z., Kushwaha P., Zoch M.L., Tomlinson R.E., Da H., Aja S., Noh H.L., Kim J.K. (2017). Sclerostin influences body composition by regulating catabolic and anabolic metabolism in adipocytes. Proc. Natl. Acad. Sci. USA..

[192] Fairfield H., Falank C., Harris E., Demambro V., McDonald M., Pettitt J.A., Mohanty S.T., Croucher P., Kramer I., Kneissel M. (2018). The skeletal cell-derived molecule sclerostin drives bone marrow adipogenesis. J. Cell Physiol..

[193] Zhu M., Liu C., Li S., Zhang S., Yao Q., Song Q. (2017). Sclerostin induced tumor growth, bone metastasis and osteolysis in breast cancer. Sci. Rep..

[194] Kaveh S., Hosseinifard H., Ghadimi N., Vojdanian M., Aryankhesal A. (2020). Efficacy and safety of romosozumab in treatment for low bone mineral density: A systematic review and meta-analysis. Clin. Rheumatol..

